# Surveillance during pregnancy: methods and response rates from a hospital based pilot study of the Pregnancy Risk Assessment Monitoring System in Ireland

**DOI:** 10.1186/1471-2393-13-180

**Published:** 2013-09-25

**Authors:** Linda M O’Keeffe, Patricia M Kearney, Richard A Greene

**Affiliations:** 1 National Perinatal Epidemiology Centre, Department of Obstetrics and Gynecology, 5th Floor, Cork University Maternity Hospital, Wilton, Cork, Ireland; 2 Department of Epidemiology and Public Health, University College Cork, Cork, Ireland

**Keywords:** Surveillance, Maternal, Pregnancy, Behaviour, Measurement tool

## Abstract

**Background:**

Many European countries including Ireland lack high quality, on-going, population based estimates of maternal behaviours and experiences during pregnancy. PRAMS is a CDC surveillance program which was established in the United States in 1987 to generate high quality, population based data to reduce infant mortality rates and improve maternal and infant health. PRAMS is the only on-going population based surveillance system of maternal behaviours and experiences that occur before, during and after pregnancy worldwide.

**Methods:**

The objective of this study was to adapt, test and evaluate a modified CDC PRAMS methodology in Ireland. The birth certificate file which is the standard approach to sampling for PRAMS in the United States was not available for the PRAMS Ireland study. Consequently, delivery record books for the period between 3 and 5 months before the study start date at a large urban obstetric hospital [8,900 births per year] were used to randomly sample 124 women. Name, address, maternal age, infant sex, gestational age at delivery, delivery method, APGAR score and birth weight were manually extracted from records. Stillbirths and early neonatal deaths were excluded using APGAR scores and hospital records. Women were sent a letter of invitation to participate including option to opt out, followed by a modified PRAMS survey, a reminder letter and a final survey.

**Results:**

The response rate for the pilot was 67%. Two per cent of women refused the survey, 7% opted out of the study and 24% did not respond. Survey items were at least 88% complete for all 82 respondents. Prevalence estimates of socially undesirable behaviours such as alcohol consumption during pregnancy were high [>50%] and comparable with international estimates.

**Conclusion:**

PRAMS is a feasible and valid method of collecting information on maternal experiences and behaviours during pregnancy in Ireland. PRAMS may offer a potential solution to data deficits in maternal health behaviour indicators in Ireland with further work. This study is important to researchers in Europe and elsewhere who may be interested in new ways of tailoring an established CDC methodology to their unique settings to resolve data deficits in maternal health.

## Background

Maternal behaviours and experiences around the time of pregnancy have a significant impact on both the short and long term health and wellbeing of mother and infant. Modifiable determinants of preterm birth and low birth-weight in developed countries include cigarette smoking during pregnancy [1] and high body mass index (BMI) [2]. A significant proportion of other adverse pregnancy outcomes such as pre-eclampsia are also associated with pre-existing maternal conditions such as high BMI and high blood pressure that are modifiable by maternal behaviour change [3]. Research has shown that these conditions are not only a significant cause of morbidity and mortality at birth but are associated with adverse health outcomes throughout the life course [4]. Low birth weight infants are at increased risk of high blood pressure in adulthood [5], type 1 [6] and type 2 diabetes [7], overweight [8], and all-cause mortality including death from cancer and heart disease [9]. Women diagnosed with pre-eclampsia are often at increased risk of future cerebrovascular or cardiovascular events [10]. Consequently, surveillance of behaviour and experiences during pregnancy is essential for improving current maternal and infant health as well as long term population health.

Across Europe on-going, timely and nationally representative surveillance of behaviors and experiences that occur during pregnancy is lacking [11]. The European Peristat Project which collated national data from over 26 European countries including Ireland on available health, social and clinical characteristics of women giving birth in 2004 illustrated perinatal data deficits in many countries [11]. Limited socio-demographic and clinical data were provided as a by-product of routine data collection such as civil registration, hospital discharge data and medical birth registries [11,12]. This was supplemented by data from national cross sectional or panel surveys examining health behaviours such as smoking during pregnancy which provided sparse and intermittent population based estimates on maternal and infant health for comparison.

Establishing on-going, surveillance based data collection which monitors changes in important pregnancy experiences and behaviors that can often only be obtained through maternal self-report, at a national and European level, in a standardised and systematic manner is essential for health policy and program development. This type of surveillance has been conducted in the United States since 1987 when the Pregnancy Risk Assessment Monitoring System (PRAMS) was established to monitor the experiences and behaviors of women before, during and after pregnancy [13]. PRAMS is now in its 36th year and is operational in over 40 states and New York City [13]. PRAMS is unique in its ongoing, population based, standardized, data to action driven approach. It uses core questions which are administered 2-4 months after pregnancy via postal survey to ongoing monthly samples of women in all PRAMS States to allow for reliable and appropriate comparisons between states and over time [14]. However, the system is also highly flexible, facilitating the addition of a selection of other data points which address state level data needs [14].

PRAMS surveillance in the United States has been used to monitor trends in maternal behaviors and experiences around the time of pregnancy, plan programs and policies, enact legislation and reduce health inequalities for population health improvement [15]. The PRAMS system has been responsible for significant maternal and infant health advancements in the United States since 1987. Examples include examining the impact of state breastfeeding law on breastfeeding rates [16], development of programs and legislation to reduce high unintended pregnancy rates in Georgia, Washington and Oklahoma [17] and monitoring achievement toward Healthy People objectives around multivitamin use, smoking and physical abuse during pregnancy [15]. The data to action driven approach of PRAMS surveillance is an initiative which may be adaptable to the European context for policy and program development in maternal and infant health.

The aim of this study was to assess the feasibility and validity of implementing a modified PRAMS methodology in Ireland. The specific objectives included

i) assessment of feasibility through development and implementation of a PRAMS Ireland survey and protocol and examination of response rates and characteristics associated with response

ii) assessment of validity through examination of item completeness, comparison of participant demographics with hospital and national birth characteristics and comparison of prevalence estimates with the nationally representative GUI [Growing up in Ireland] cohort.

## Methods

### Sampling strategy

The standard approach to sampling in all 40 US states in which PRAMS is in operation involves use of state birth certificate files to generate stratified, random samples of women with recent live births [14]. However, in Ireland, access to the Irish birth certificate file is restricted under the Data Protection Act 1988 [18]. Consequently, Cork University Maternity Hospital (CUMH), a large urban obstetric unit in the south of Ireland delivering almost 9,000 babies per year [19] or approximately 12% [20] of all Irish births was chosen to test the PRAMS methodology.

Using a sampling frame of live births recorded 3 – 5 months before the study start date at CUMH, 124 women were randomly sampled using a random start and constant sampling fraction. The sample size for the pilot was based on methods recommended by Thabane et al., 2010 [21] using a confidence interval approach to estimate sample sizes for pilot studies. To estimate a projected response of 65% [based on CDC minimum weighted response rates] with a lower confidence limit of 60% and upper confidence limit of 75% we sampled 124 women to the study. Women were included by manually counting and extracting a record from each of two delivery books, one recording caesarean sections and the other recording both spontaneous and instrumental vaginal births. In line with the PRAMS protocol mothers of stillbirths, neonatal deaths and triplets or more were excluded [14]. This was done using the APGAR score recorded on the delivery record and cross-checking with hospital records for the period. Sampling was done proportionally in a ratio of 1:4 to represent the underlying proportion of caesarean deliveries in the population of approximately 25% [22]. Available information including name, address, maternal age, infant sex, gestational age at delivery, delivery method, APGAR score and birth weight were extracted from delivery records of selected women.

### PRAMS protocol and study materials

Letters and information sheets were prepared explaining the purpose of the study and its importance to improving maternal and child health in Ireland. The PRAMS survey covers a range of topics including exposures during pregnancy such as alcohol and smoking, care received, and socio-demographic information [23].

For this study, questions were carefully modified for content, language and overall layout. Content which pertained specifically to the United States such as Medicaid status and enrolment in the Women, Infants and Children Supplemental Nutrition Programme (WIC) were removed and replaced with medical card and private health insurance questions relevant to Ireland. Language changes were applied to conform to commonly used terms in Ireland such as; “antenatal” rather than “prenatal”; “contraception” rather than “birth control” and “health care professional” rather than “health care worker”. Demographic information including race/ethnicity, marital status, nationality, educational attainment and health insurance status provided by the birth certificate file in the United States were added to the questionnaire, as these were not available from the CUMH delivery record. Validated questions from other longitudinal or cross sectional studies were used to supplement the PRAMS questionnaire or replace questions where necessary including questions on pregnancy history from the Avon Longitudinal Study of Parents and Children (ALSPAC) [24], questions on complications during pregnancy from GUI [25] and Growing up in Australia [26] and questions on sexually transmitted infections from The Irish Survey of Sexual Health and Relationships (ISSHR) [27]. These questions were chosen over some PRAMS Phase 6 questions for comparability.

Recent research suggests that the ascertainment of alcohol exposure during pregnancy in studies focused on documenting patterns of alcohol consumption during gestation could be optimized by examining more carefully, the dose, pattern and timing of exposure [28]. Moreover, the most recently available estimates in the Irish general population suggest that up to 77% of women regularly drink alcohol in Ireland compared to an EU average of 68% while over 42% of all female drinkers are classified as having harmful drinking patterns [29]. As a result, we developed questions on maternal alcohol consumption specific to these needs for PRAMS in Ireland which took into account the dose, pattern and timing of alcohol exposure during pregnancy based on work by O’Leary et al., 2010 [28].

Diet around the time of pregnancy has a substantial impact on maternal and infant health but remains one of the key determinants of health that has never been addressed in PRAMS in the United States. PRAMS does not collect data on diet potentially due to feasibility issues and the impact on response. Furthermore, little is known about effective approaches by which dietary data can be collected in PRAMS. Thus, we chose to randomize participants to receive a validated semi-quantitative Food Frequency Questionnaire [FFQ] to collect information on maternal diet in order to assess its impact on response rates. Random allocation was achieved using a random number generator in Microsoft Excel. For all 124 women, a random number between 0 and 1 was generated. The 50% of women with the highest numbers were selected to receive the validated FFQ. This FFQ was previously adapted from the European Prospective Investigation of Cancer (EPIC) study [29], validated in the Irish general population [30] and used in the Survey of Lifestyle Attitudes and Nutrition (SLAN) 1998, 2002 and 2007 [29] in the Irish general population.

The layout and design features of the questionnaire were also adjusted to incorporate the most recent evidence from a Cochrane Systematic Review on improving response rates to postal questionnaires [31]. This involved changing PRAMS questions to a horizontal rather than vertical orientation which has been shown to increase response rates [31].

This research protocol and all study materials administered within this study received ethical approval from the Cork Research Ethics Committee of the Cork Teaching Hospitals (CREC).

### Data analysis

Statistical analyses were conducted in STATA V. 11. Descriptive statistics including mean for continuous variables and frequencies for categorical variable were used to examine response rates, characteristics associated with response and non-response as well as missing data. We compared available demographic and clinical information from the delivery record at CUMH between responders and non-responders using a Pearson *χ*^2^ [chi] squared test for the difference in proportions. Respondents were defined as eligible women who were selected to receive a survey, and completed the survey within two months of the study start date. Demographic characteristics of respondents were also compared to recently available data for CUMH deliveries for 2010 [19] and data on all births in Ireland for 2010 [22]. Rates of missing data for each question were calculated to examine the completeness of response per survey item. The average number of days to response from receipt of the first survey was also calculated. The prevalence of behaviours and experiences reported in the PRAMS study were also compared to findings from the GUI, a longitudinal study conducted in Ireland between 2008-2009 among mothers of live births, approximately 9 months after birth [32]. GUI was chosen for comparison as it was nationally representative of all live births in 2009.

## Results

**Figure 1:**
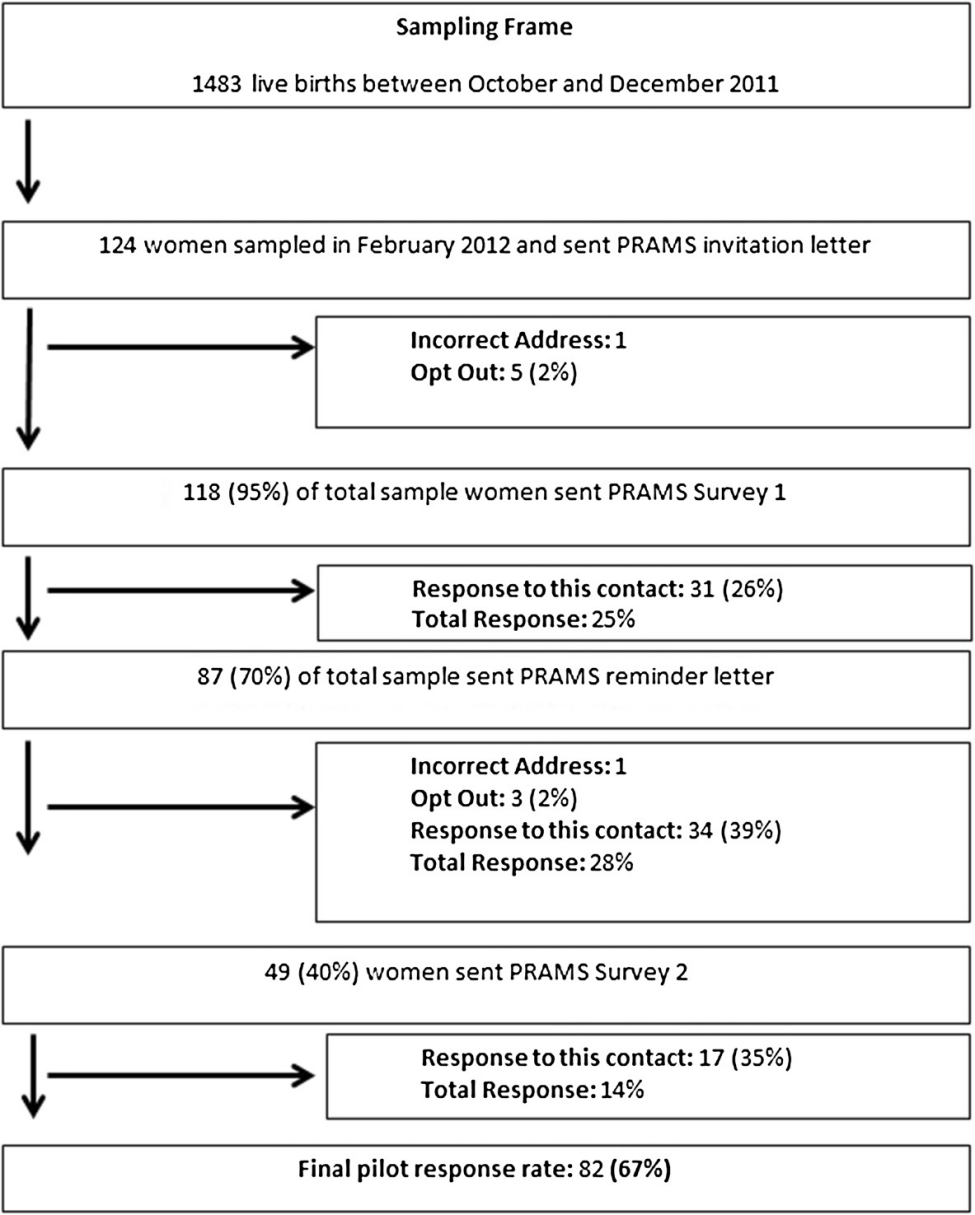
PRAMS protocol.

The PRAMS protocol implemented is shown in Figure 1. The total pilot cost was $3500 dollars: $1000 for design costs for the PRAMS questionnaire, $1500 for printing and postage costs and $1000 for labor costs including study management and packaging. In total, 67% of sampled women responded to the PRAMS survey. The average number of days to response from distribution of the first survey was 18. The reminder letter contributed the highest proportion to the overall response rate. After the second survey was administered, no further contact with women was made as the response rates had exceeded 65%, the minimum weighted response rate for PRAMS recommended by the CDC [33]. A comparison of characteristics of PRAMS respondents to women delivering in CUMH in 2010 or nationally in 2010 is shown in Table 1. PRAMS respondents had a higher proportion of Irish and married women than CUMH or all national singleton deliveries in 2010. PRAMS also reflected a higher proportion of caesarean delivered women and preterm births than either the CUMH population or national deliveries in 2010. Overall, women who did not respond were slightly younger than respondents and had a higher proportion of female babies, caesarean sections and preterm birth (Table 2).

**Table 1:** Demographic characteristics by type of contact in PRAMS pilot study

	**PRAMS n=82 n (%)**	**CUMH deliveries n=8,898 n (%)**	**National deliveries n=72,709 n (%)**
**Age[mean, (SD)]**	33 (5)	*No data*	31 (5)
**Nationality**			
Irish	71 (86.6)	6,998 (80.3)	54,684 (75.2)
Other*	11 (13.4)	1,714 (19.7)	18,025 (24.8)
**Married**	72 (87.8)	*No data*	47,528 (65.4)
**Primiparous**	30 (36.6)	3,604 (41.4)	30,099 (41.4)
**Education**			
Second Level	16 (19.5)	*No data*	*No data*
Third Level**	66 (80.5)	*No data*	*No data*
**Delivery Mode**			
Spontaneous vaginal	43 (52.4)	4,686 (52.7)	41,946 (57.8)
Other vaginal***	10 (12.2)	1,659 (18.6)	12,029 (16.5)
**Total Vaginal**	53 (64.6)	6,345 (71.3)	53,975 (74.3)
Elective	15 (18.3)	1,302 (14.9)	*No data*
Emergency	4 (4.9)	1,138 (13.1)	*No data*
**Total Caesarean**	19 (23.1)	2,440 (28.0)	20,373 (27.0)
**Low birth weight**	4 (4.9)	463 (5.2)	2,598 (3.6)

*Other includes Non-Irish EU National, Other White Background, African, Any Other Black Background, Chinese and Any Other Asian Background.

**Any third level qualification including Diploma/Certificate, Primary Degree or Postgraduate/Higher Degree.

***Ventouse, forceps, combined instrumental, vaginal breech.

**Table 2:** Characteristics of PRAMS sample to available demographics for responders and non-responders

	**PRAMS sample n=124 n (%)**	**Responders n=82 n (%)**	**Non-responders n=42 n (%)**	**P value**
**Age mean, (SD)**	32 (5)	33 (5)	31 (5)	0.050
**Infant Gender**				
Female	62 (50)	36 (43.9)	26 (61.9)	0.076
Male	60 (48.4)	44 (53.7)	16 (38.1)	
**Delivery Mode**				
Vaginal	83 (76.9)	61 (74.4)	27 (64.3)	0.928
Caesarean Section	41 (33.1)	21 (25.6)	14 (33.3)	
**Birth weight** (<2500g)	6 (4.8)	2 (2.4)	4 (9.5)	P<0.01
**Preterm birth** (<37 weeks gestation)	8 (6)	4 (4.9)	4 (7.7)	0.319

*p value is for the difference in proportions for categorical variables from *χ*^2^ test or difference in means for continuous variables from t tests between responders and non-responders.

Table 3 shows minimum completion rates for all survey questions per section of the PRAMS pilot survey. Demographic data, labour and delivery questions and the period since the baby was born were best completed which allowed for almost complete socio-demographic data for PRAMS respondents.

**Table 3:** Minimum completion rate for survey items on the PRAMS survey

**Section**	**Category**	**No of survey items**	**Completeness rate**
**Section A**	Demographics	11	98%
**Section B**	Pregnancy History & Before Pregnancy	97	92%
**Section C (i)**	During Pregnancy	64	90%
**Section C (ii)**	Alcohol Use in Pregnancy	21	93%
**Section E**	Antenatal Care	61	72%
**Section F**	Labour and Delivery	20	99%
**Section G**	Time Since Baby was Born	88	94%
	**Total**	362	91%

PRAMS respondents reported a low prevalence of smoking during pregnancy, high prevalence of folic acid intake before and during pregnancy, higher use of assisted reproductive technologies and high rates of ever having breastfed compared to participants in the GUI study (Table 4). The respondents also reported a high prevalence of alcohol use before and during pregnancy. The prevalence of nearly all complications or conditions associated with pregnancy was higher than those reported by GUI participants.

**Table 4:** Prevalence of selected behavioural characteristics in the PRAMS study and estimates from GUI 2010

	**PRAMS n=82 n (%)**	**GUI n=10,953 n (%)**
**Smoking status**		
Ever smoked	47 (57.3)	3,078 (37.9)
Smoked in pregnancy	6 (7.3)	1,973 (18)
Current Smoking	12 (14.6)	2,798 (25.6)
**Alcohol Consumption**		
Consumed alcohol before pregnancy	60 (73.2)	9,185 (83.8)
Consumed alcohol in pregnancy	43 (52.4)	2,164 (19.7)
Consumed alcohol (1st Trimester)	23 (28.1)	1,100 (10.1)
Consumed alcohol (2nd Trimester)	35 (42.7)	1,544 (14.1)
Consumed alcohol (3rd Trimester)	30 (36.6)	1,513 (13.8)
**Folic Acid Use**		
Used folic acid before pregnancy	60 (73.2)	6,861 (63.8)
Used folic acid in the first trimester	80 (97.7)	10,760 (93.4)
**Pregnancy intention: wanted to be pregnant**	43 (52.4)	6,276 (58.4)
**Ever Breastfed**	54 (65.9)	6,116 (55.9)
**Care Received/Service Use**		
Used assisted reproductive technologies	7 (8.5)	456 (4.2)
Admission to Neonatal Unit	7 (8.5)	1,574 (14.4)
Shared care (hospital and general practitioner)	62 (75.6)	8,378 (77.8)
**Complications during pregnancy**		
Any complication	43 (52.4)	5,943 (54.3)
Nausea/vomiting	11 (13.4)	1,914 (17.5)
Urinary Tract Infection	15 (18.3)	1,589 (14.5)
Raised blood pressure	14 (17.1)	1,196 (10.9)
Pre-eclampsia	7 (8.5)	791 (7.2)
Gestational diabetes (diet)	6 (7.3)	245 (2.2)
Gestational diabetes (insulin)	3 (3.7)	104 (1.0)
Bleeding	12 (14.6)	645 (5.9)
Placenta Praevia	3 (3.7)	305 (2.8)

## Discussion

The results of this pilot study show that it is feasible to administer a modified PRAMS questionnaire [23] and protocol in Ireland. Previous population based postal surveys in the Irish general population such as the SLAN surveys administered in 1998, 2002 have achieved response rates of 62% and 53% respectively [29]. The final response rate of the GUI Study [34], a survey administered face to face by trained interviewers in six different languages in a population of over 10,000 mothers in 2008 was 70%. Our response rate of 67% compares favourably with these and shows the validity of the study materials and methodology. In addition low opt out rates (7%) and high item completion rates indicate that the PRAMS materials and protocol implemented were well received. Our response rate exceeded the CDC minimum response rates for PRAMS of 65% without a third mail survey, a telephone follow-up, rewards or incentives which are included routinely in the United States [14].

The prevalence of behaviours and experiences collected in PRAMS is comparable to some recent population representative estimates from GUI. Overall, PRAMS may have over-represented married, Irish, educated women [80% had a third level education compared with 36% nationally based on SLAN 2006 [29]. This may explain the higher prevalence of protective health behaviours such as folic acid intake and breastfeeding. In relation to the higher reported prevalence of alcohol use in pregnancy, our data are more comparable with prevalence estimates from the United Kingdom [35] and the Netherlands [36]. It is possible that we may have obtained more reliable estimates of alcohol use due to anonymised postal data collection, which has been shown to obtain more reliable responses on socially un-desirable behaviours such as alcohol use [37]. We also found a higher prevalence of pregnancy conditions and complications which may be the result of improved recall compared to the participants of GUI, who were sampled between 9 months and 1 year postpartum.

Although the use of one large hospital is a potential limitation of our work, as almost 99% of Irish births occur in Irish maternity hospitals, hospital based sampling does provide almost complete coverage of recent live births in Ireland including under-served or disadvantaged groups, thus potentially allowing health disparities to be addressed. In addition as births in this unit represented 12% of all Irish births or almost 2/3 of all births in the health services region we suggest that this pilot study could be broadly representative of the feasibility and validity of PRAMS in the hospital system in Ireland. However, though the pilot reveals PRAMS to be feasible and a potentially valid data collection tool for maternal behaviour surveillance in Ireland, other hospitals considering this sampling strategy may find the paper based approach used to be inefficient and time consuming if implemented as a routine data collection system particularly if done on a larger scale. Furthermore, record systems in each of the 20 Irish maternity hospitals vary substantially and thus it may be difficult to replicate exactly the protocol implemented here.

A potentially more efficient hospital based approach which overcomes some of the challenges faced in this pilot includes the use of a national patient electronic record to sample women through the National Maternal Newborn Clinical Management System due to replace the current paper based record system across Irish maternity hospitals in 2014. This approach would potentially allow for automated, stratified, random sampling at a national level which minimises data extraction error at the point of sampling. Moreover, it would provide reliable information on baseline characteristics of sampled participants, provide a sampling frame complete for almost 99% of births in Ireland and allow over-sampling of vulnerable population groups which are under-represented in this pilot study. With the development and roll out of this system in 2014, an on-going hospital based PRAMS surveillance system with a capacity for over-sampling of minority population groups to address health disparities in Ireland could be feasible.

## Conclusions

The PRAMS surveillance system is a unique behavioural surveillance initiative around the time of pregnancy which may offer a potential solution for European countries experiencing deficits in high quality, population based data on maternal behaviours and experiences during pregnancy which can only be obtained through maternal self-report. The results of this study show that the PRAMS methodology is a feasible and valid approach to collecting information on maternal experiences and behaviours in Ireland. The extent to which the materials administered in the pilot study were well received highlights the adequacy of the modified study instruments and protocol for a full scale PRAMS surveillance system. The strong willingness to participate in the pilot study would be indicative of potentially high response rates in an on-going hospital based surveillance project in Ireland. The distribution of response rates by type of contact reveals the potential effectiveness of the numerous and frequent contacts in the Irish context. The prevalence estimates obtained for many behaviours shows participants willingness to report on socially undesirable behaviours such as alcohol use during pregnancy. High item completion rates illustrate the effectiveness of both the design of the survey and questions included at capturing valid and complete responses from participants. However, lower completion rates in antenatal care are a limitation and this must be addressed in design, layout and content revisions of the survey given the overall aims and objectives of PRAMS. Further work is now required to expand this approach for a nationwide surveillance effort across all hospitals potentially using the new Maternal Newborn Clinical Management System. This would allow for efficient on-going data collection, complete coverage of all live births in Ireland and stratification or over-sampling among socially disadvantaged groups of women who are less likely to respond and more likely to be experiencing health disparities.

### Summary

• PRAMS is the only on-going, population based surveillance system of maternal behaviours and experiences before, during and after pregnancy worldwide.

• Many European countries lack reliable, on-going, population based data on maternal behaviours and experiences around the time of pregnancy.

• PRAMS is a feasible and valid approach to surveillance of behaviours and experiences during pregnancy in Ireland.

• This study is important to maternal and child health researchers in Europe or elsewhere who may be interested in new ways of adapting an established CDC methodology to their own unique settings to build data capacities for policy and program development.

## Competing interests

The authors declared that they have no competing interests.

## Authors’ contributions

LMOK completed data analysis and prepared the draft manuscript. PMK and RAG edited the manuscript. All authors approved the final version for publication. The authors do not have any conflicts of interest. The data has not been published previously and it not under consideration for publication elsewhere.

## Pre-publication history

The pre-publication history for this paper can be accessed here:

http://www.biomedcentral.com/1471-2393/13/180/prepub
